# Supplementary figures and images for: Safety and Efficacy of Felid Herpesvirus-1 Deletion Mutants in Cats
Source: Viruses. 2021 Jan 22;13(2):163. doi: 10.3390/v13020163 (PMC7911815; doi:10.3390/v13020163)

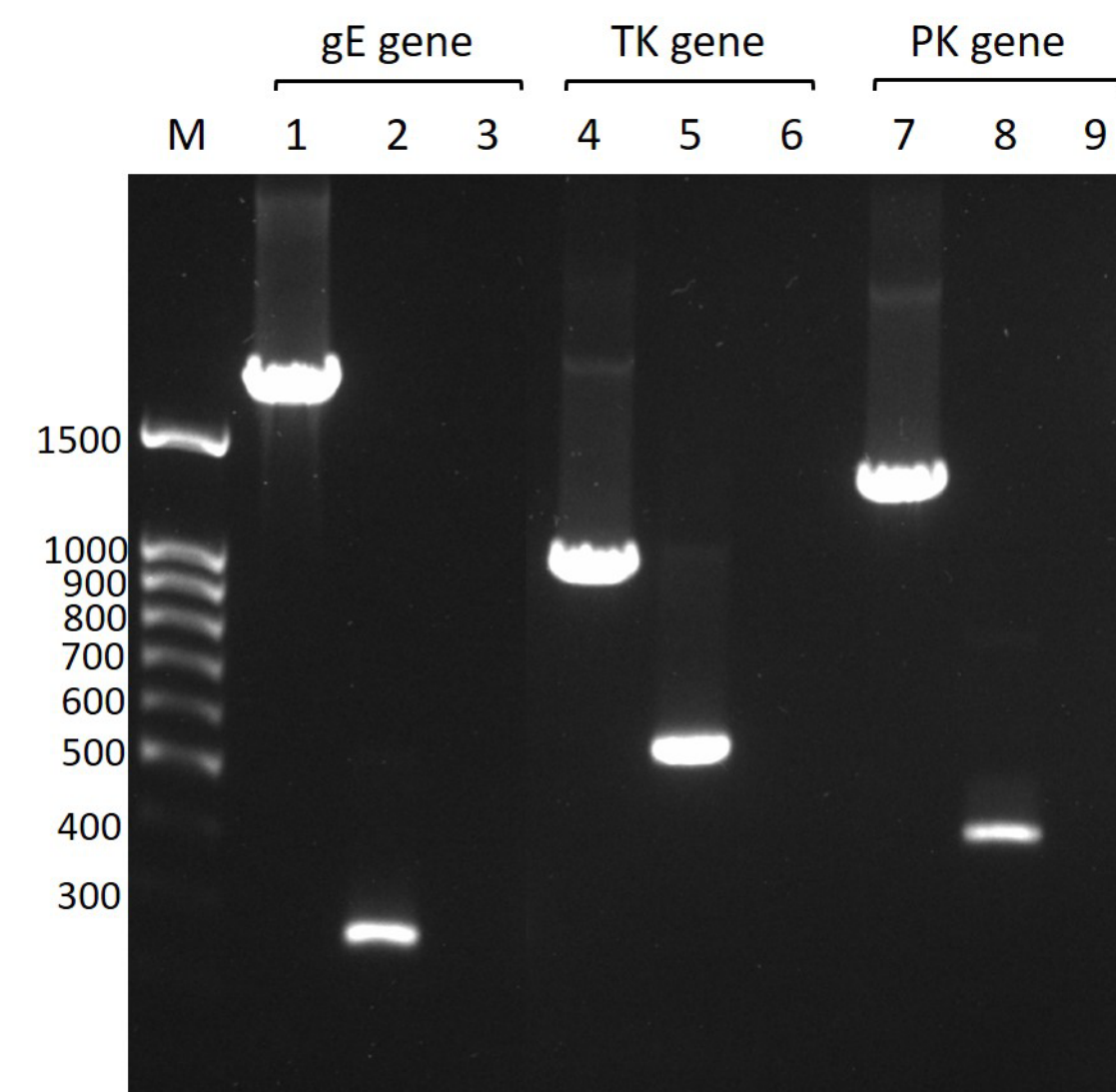

Supplemental Figure S1

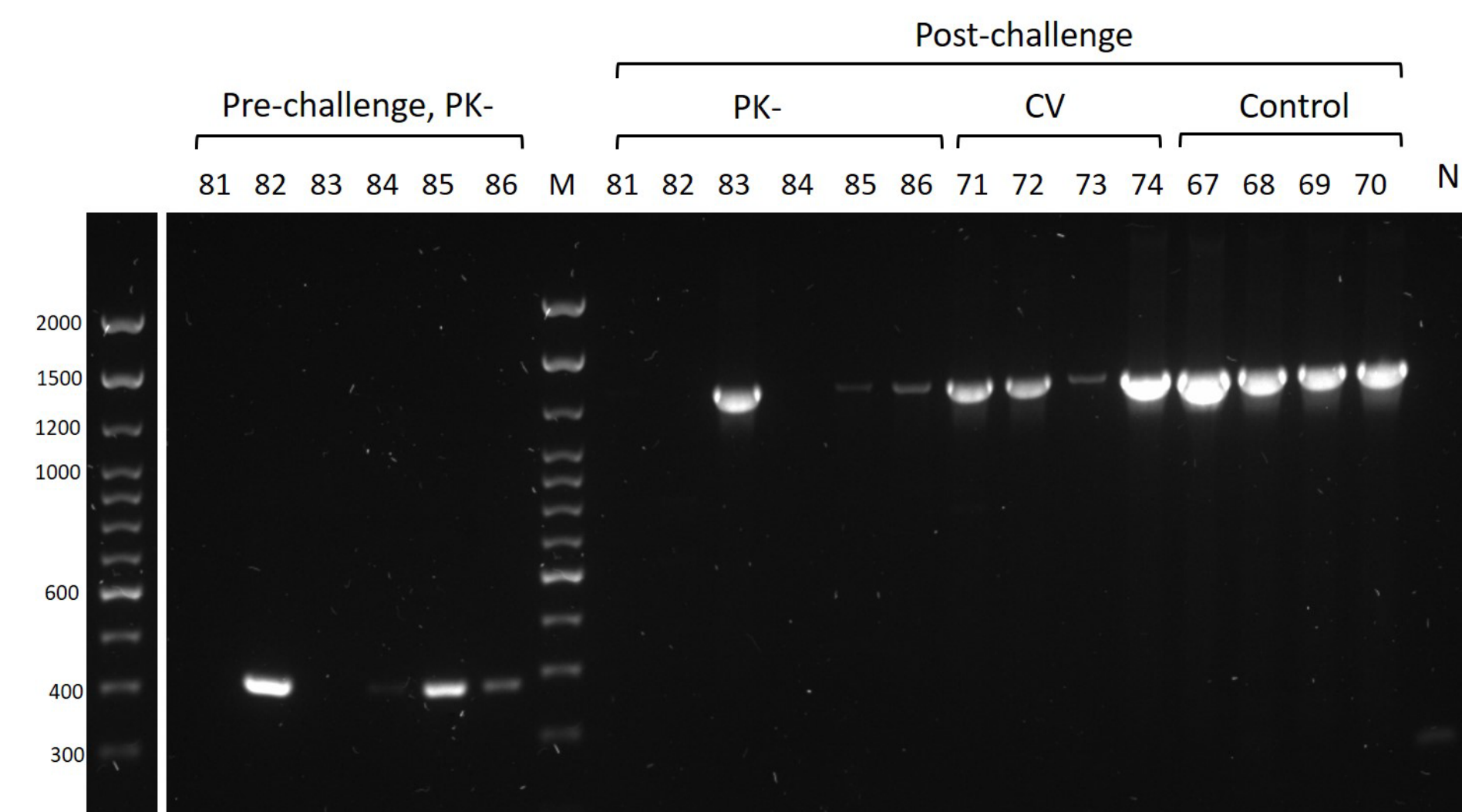

Supplemental Figure S2

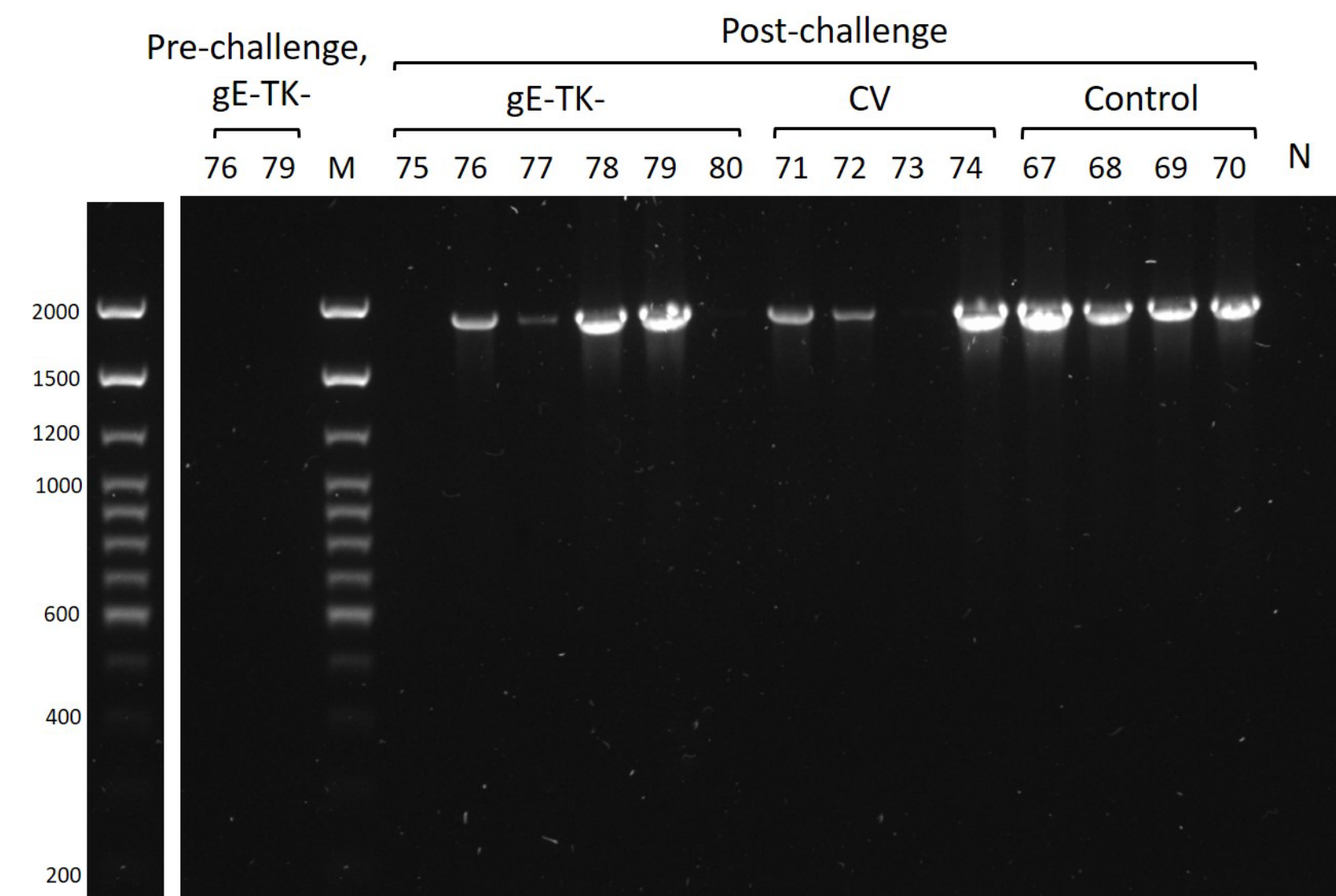

Supplemental Figure S3

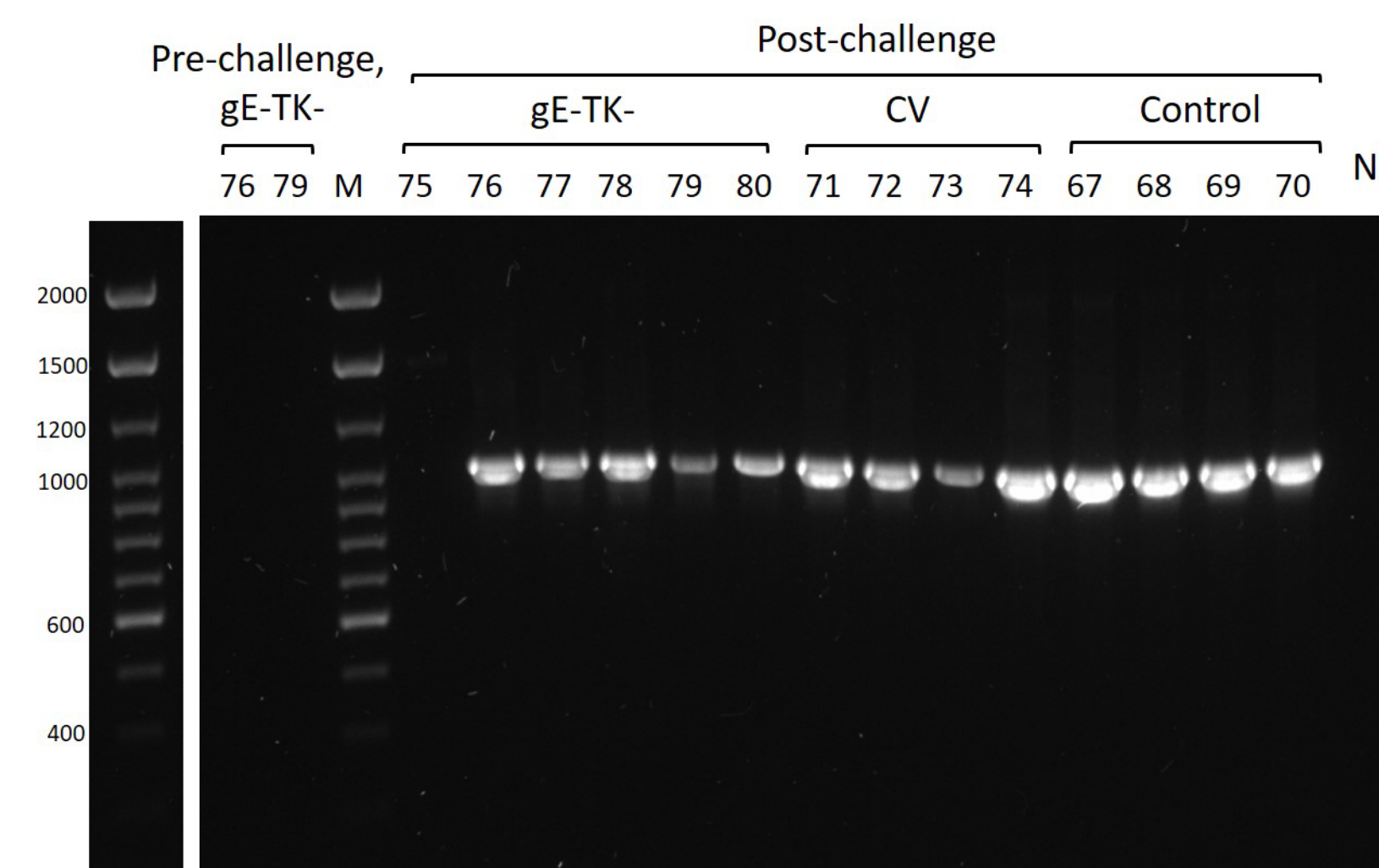

Supplemental Figure S4

Supplement: Supplementary file 1 [file viruses-13-00163-s001.pdf]
